# Lack of involvement of CD63 and CD9 tetraspanins in the extracellular vesicle content delivery process

**DOI:** 10.1038/s42003-023-04911-1

**Published:** 2023-05-17

**Authors:** Maria Laura Tognoli, Julia Dancourt, Emeline Bonsergent, Roberta Palmulli, Olivier G. de Jong, Guillaume Van Niel, Eric Rubinstein, Pieter Vader, Gregory Lavieu

**Affiliations:** 1 CDL Research, University Medical Center Utrecht, Utrecht University, Utrecht, The Netherlands; 2 Université Paris Cité, INSERM U1316, UMR 7057/CNRS, Paris, France; 3 Institute of Psychiatry and Neuroscience of Paris (IPNP), INSERM U1266, Paris, France; 4 Department of Pharmaceutics, Utrecht Institute of Pharmaceutical Sciences, Utrecht University, Utrecht, The Netherlands; 5 Sorbonne 5 Université, INSERM, CNRS, Centre d’Immunologie et des Maladies Infectieuses, CIMI-Paris, Paris, France; 6 Department of Cardiology, Experimental Cardiology Laboratory, University Medical Center Utrecht, Utrecht University, Utrecht, The Netherlands

**Keywords:** Cell biology, Biochemistry

## Abstract

Extracellular vesicles (EVs) are thought to mediate intercellular communication by transferring cargoes from donor to acceptor cells. The EV content-delivery process within acceptor cells is still poorly characterized and debated. CD63 and CD9, members of the tetraspanin family, are highly enriched within EV membranes and are respectively enriched within multivesicular bodies/endosomes and at the plasma membrane of the cells. CD63 and CD9 have been suspected to regulate the EV uptake and delivery process. Here we used two independent assays and different cell models (HeLa, MDA-MB-231 and HEK293T cells) to assess the putative role of CD63 and CD9 in the EV delivery process that includes uptake and cargo delivery. Our results suggest that neither CD63, nor CD9 are required for this function.

## Abstract (web summary)

In human cell lines, CD63 and CD9 tetraspanins are knocked-down and shown to not be required for extracellular vesicle uptake and cargo delivery.

## Introduction

Extracellular vesicles (hereafter named EVs) serve as intercellular communication vectors and are involved in a broad range of physiological functions. EVs are thought to sample the content of the donor cell and mediate its transfer into acceptor cells^1^. Several groups, including ours^2–4^, demonstrated that EV uptake and content delivery occurs and can be quantified within well-established cell lines^5^.

Tetraspanin CD63 and CD9 are highly enriched in EVs^6,7^, and are, at least in specific cell types, respectively enriched within multivesicular bodies/endosomes or at the plasma membrane of cells^8^.

Interestingly, CD63 has been shown to participate in the infection of several viruses, demonstrated using mostly inhibitory antibodies or RNA interference technology. Several studies have shown that silencing CD63 reduced HIV infection, but it remains unclear if the protein is required for virus entry, or during infection and the packaging of structural viral proteins (gag and env)^9^. Other studies showed that CD63-syntenin1 complex is required for post-endocytic trafficking of papillomaviruses^10^.

Interestingly, it has been shown that Cas9-mediated KO of CD63 decreases the production of small vesicles, suggesting a role of CD63 in exosome biogenesis^11^. To our knowledge, it remains to be tested if CD63 has an impact on EV uptake and content delivery.

Equally interesting is the putative role of CD9 in the regulation of membrane fusion. CD9 is a long-time contender for the regulation of membrane fusion during myoblast formation^12^ or sperm-egg fecundation^13^, perhaps due to its capacity to induce or to be concentrated in high membrane curvature areas that favor fusion^14^. As described above for CD63, CD9 seems also involved in virus infection and has been proposed to play a role in virus entry^15^.

In addition, some studies suggested that treatment with anti-CD9 antibodies impairs internalization of EVs, suggesting a direct role of CD9 in EV-uptake^16,17^.

Therefore, since the discovery of EVs and the identification of CD63 and CD9 within those entities, both tetraspanins have been suspected to be more than EV markers by playing a direct or indirect role in the EV delivery process. This tempting hypothesis is supported by the many reports that established links between those tetraspanins and viruses entry or membrane fusion, two EV-related topics. Despite those speculations, the impact of CD63 and CD9 depletion on EV uptake has never been rigorously tested. We therefore decided here to test this simple hypothesis using independent but complementary methods that we recently developed.

Our respective groups previously established independently novel cell-based assays to characterize at the cellular and molecular levels the EV uptake and content delivery process. Together our previous results suggest that EVs are internalized mostly by macropinocytosis, and that EV cargo delivery occurs through a pH and protein-dependent mechanism that resembles fusion between EV and endosomal membranes.

Importantly, our respective assays rely on fundamentally distinct principles.

Vader’s group developed a CRISPR-Cas9-based reporter system that allows the direct functional study of EV-mediated transfer of sgRNA molecules at single-cell resolution^4^. To visualize such a transfer, they designed a fluorescent “Stoplight” reporter system which is permanently activated in EV-acceptor cells upon functional transfer of a specific targeting sgRNA, expressed in EV-donor cells. Briefly, untreated acceptor cells express a red fluorescent protein (mCherry) along with the Cas9 protein. When donor EVs deliver a sgRNA targeted against a linker region downstream of the mCherry encoding gene and before its stop codon, Cas9-mediated introduction of frameshift of either +1nt or +2nt allows bypass of the original stop codon and permanent expression of a green fluorescent protein (eGFP), along with mCherry. As a result, red acceptor cells also fluoresce in green. This system, named CROSS-FIRE, highlighted the importance of certain molecules, such as Rab5, and Rab7 known to control endosomal maturation, thus supporting our delivery model.

Here, we used the CROSS-FIRE system to test the impact of CD63 and CD9 knockdown in MDA-MB-231 donor and HEK293T acceptor cells, through siRNA technology.

Lavieu’s group chose a cargo-based approach, in which they follow the fate of isolated donor EVs loaded with nanoluciferase-tagged HSP70, a generic EV cargo^3^. They first measured the luminescence (emanating from the EV cargo) associated with the acceptor cells to bulkily assess the uptake process. EV uptake represents the sum of EV-cargo i) associated with the plasma membrane of the acceptor cell, ii) internalized within the endo/lysosomal compartments, iii) released in the cytosol. Then, by applying mechanical disruption to separate the membrane from cytosol, they acutely measured the luminescence truly delivered in the cytosol to determine the content delivery. Importantly, results can be independently confirmed by subcellular imaging, using fluorescently-tagged HSP70.

Here, we capitalize on those assays and test if EV uptake and delivery are impacted when donor or acceptor HeLa cells have been knocked-out for CD9 or CD63, using CRISPR-Cas9 technology.

In this study, we tested different cell lines (HeLa, MDA-MB-231, HEK293T), two independent assays relying on different principles, and two methods to knockdown CD63, CD9 expression in donor and acceptor cells to formally demonstrate that the expression of CD9 and CD63 is not required for EV uptake and content delivery.

## Results

**Fig. 1:**
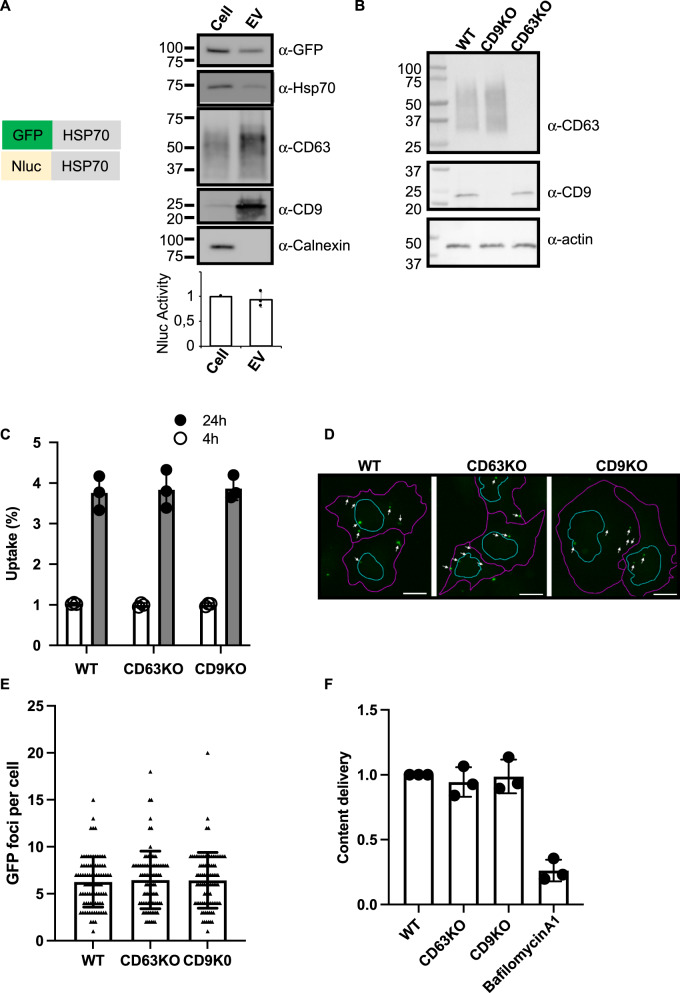
Impact of CD9/CD63 depletion on acceptor cells using the NLuc assay. **A** Schemes of the chimeric EV-cargoes used for fluorescence- and luminescence-based assays. EV characterization by western blot. Equal amounts of protein from cell lysate or isolated HeLa EVs were tested by western blot for endogenous HSP70, CD63, CD9 and Calnexin. GFP was also tested for GFP-HSP70-expressing cells. Graph shows the luminescence activity of NLuc-HSP70 in cell lysate and EVs (equal amounts of protein). Activity in cell lysate was set to 1. Three independent measurements were performed. **B** Immunoblots showing the expression of CD63, CD9 and actin (loading control) in WT (control), CD63KO and CD9KO HeLa cells. **C** Graph showing the EV-uptake (% from the input) in WT, CD63KO and CD9KO acceptor cells. NLuc-HSP70 positive EVs were loaded on acceptor cells for 4 (white bar) and 24 h (grey bar), prior to luminescence detection. Three independent experiments were performed, each including three replicates. Each dot represents the mean of a triplicate. Errors bars represent the standard deviations of the means. **D** Confocal micrographs showing WT, CD63KO and CD9KO acceptor cells treated with GFP-HSP70 positive EV for 24 h. Cells were fixed to monitor internalized GFP-HSP70 EVs (white arrows). Purple lines represent cell outline and cyan lines represent nucleus outline. Scale bar, 10 μm. **E** Graph showing number of GFP foci per GFP positive cells. Three experiments were performed and 88 cells were tested per condition. Each dot represents a cell, error bars represent the standard deviations. **F** Graph representing the content delivery within the cytosol of WT, CD63KO and CD9KO acceptor cells. 24 h after treatment with NLuc-HSP70 positive EVs, acceptor cells were mechanically disrupted to separate membrane and cytosolic fractions, and measure the % the luminescence delivered to the cytosol. Value in WT cells (control) was set to 1. Three independent experiments were performed, each dot represents an experiment. Error bars represent the standard deviations.

We first isolated by sequential ultracentrifugation, donor EVs containing NLuc-HSP70, a generic EV cargo fused to a luminescent reporter, from HeLa cells stably expressing the chimeric protein. Consistently with our previous studies^2,3^, EV isolated by sequential ultracentrifugation were enriched in classical EV markers (CD63, CD9) and depleted from others intracellular proteins such as Calnexin, a marker of endoplasmic reticulum (Fig. 1A). Importantly, our chimeric cargo was found in the isolated EVs.

To test our hypothesis we used, as acceptor cells, WT HeLa cells, or HeLa knocked-out for CD63 or CD9, using CRISPR technology. Efficiency of the Cas9-mediated KO was confirmed by immunoblot (Fig. 1B).

Isolated vesicles containing the luminescent reporter were then incubated with acceptor cells for short or long time (4 and 24 h, respectively). Acceptor cells were washed to remove vesicles that remained in the media, and we measured the luminescence emanating from the EV-cargo to assess EV uptake (Fig. 1C). Note that EV uptake may include signal emanating from EVs bound to the cell surface, internalized EVs, or EV-cargo that has been released in the cytosol of the acceptor cells. No differences were observed between the control and cells depleted of CD63 or CD9. In both cases, the luminescent signal increased over time, reaching around 4% (of the total EV input) after 24 h, consistently with our previous results. To localize EVs within acceptor cells, we replaced the luminescent EV cargo with GFP-tagged HSP70. We observed several GFP-positives foci within all WT or KO acceptor cells, suggesting EV internalization within the endosomal compartment, as previously described (Fig. 1D). Quantifying the number of foci per GFP-positive cells did not reveal any differences between control cells and CD63/CD9 KO cells (Fig. 1E). Altogether these results suggest that the uptake of HeLa EVs, and more precisely the internalization of EVs, is not impacted by depletion of CD63 and CD9 within HeLa acceptor cells.

Importantly, our luminescence-based assay enables to directly measure EV cargo delivery within the cytosol of acceptor cells^3^. To do so, acceptor cells exposed to luminescent EVs containing NLuc-HSP70 were mechanically disrupted and submitted to cell fractionation to separate the membrane from cytosolic fractions. We used this procedure to assess if CD63 or CD9 were involved in EV content delivery. The amount of luminescent cargo found in the cytosolic fraction of WT, CD63KO, and CD9KO cells was similar (Fig. 1F). As a control we used bafilomycin1, which blocks endosomal acidification. Consistently with previous results, bafilomycin1 prevented EV content delivery. These results suggest that CD63 and CD9 are dispensable for EV content release that occurs in acidic endosomes.

**Fig. 2:**
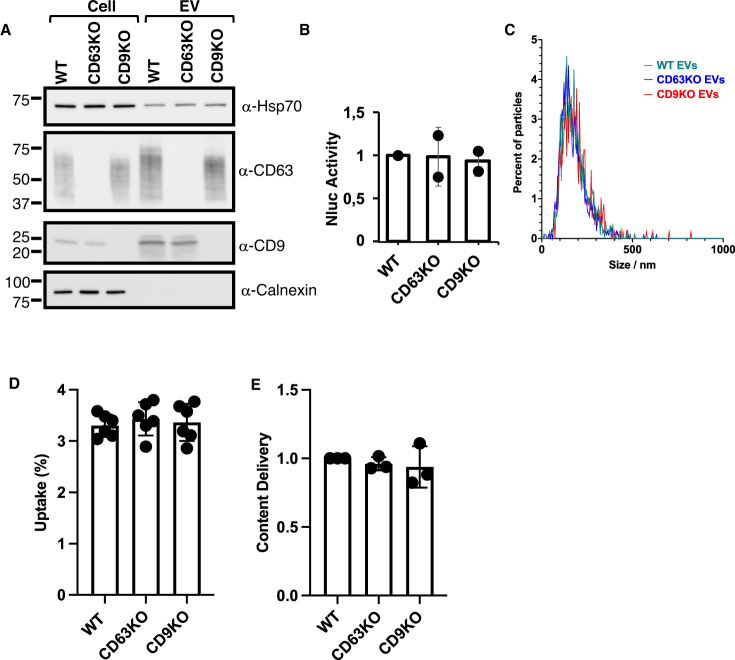
Impact of CD9/CD63 depletion on donor EVs using the NLuc assay. **A** HeLa Cell and EV contents were assessed for CD9, CD63 and Calnexin by western blot. b) WT, CD63KO and CD9KO cells were transfected with NLuc-HSP70 reporter and EVs were isolated. **B** Luminescence activity was tested in EVs emanating from the three types of donor cells (equal amount of proteins). Two independent experiments were performed in duplicate, dot represent the average of each duplicate for each experiment, error bars represent the standard deviations. **C** EVs isolated by sequential ultracentrifugation and emanating from WT, CD63KO and CD9KO cells were analyzed by nanoparticle tracking. Graphs represent the particle size distribution for each sample. **D** Graph showing the EV-uptake (% from the input) in HeLa WT cells, after incubation for 24 h with EVs emanating from WT, CD63KO and CD9KO cells. Three experiments were performed, each including duplicates. Dots represent individual replicate, error bars represent the standard deviations. **E** Graph representing the content delivery within the cytosol of WT, when EVs emanating from WT, CD63KO and CD9KO cells were added for 24 h. Value in WT cells (control) was set to 1. Three independent experiments were performed, each dot represents an experiment. Error bars represent the standard deviations.

One other possibility is that tetraspanins act on the EV side. To test this hypothesis, we compared the delivery capacity of EVs emanating from donor WT, CD63KO and CD9 KO HeLa cells. We therefore transfected donor cells with the NLuc-HSP70 reporter. After EV isolation, we roughly characterized EVs for size and protein markers. Western blots confirmed the absence of CD9 and CD63 from EVs emanating from CD9KO and CD63KO cells, respectively (Fig. 2A). CD9 and CD63 were found at comparable level when CD63KO and CD9KO derived EVs were respectively compared with WT-derived EVs. Other classical markers such as endogenous HSP70 were found in all samples at similar levels, whereas, as expected, Calnexin was absent. Specific activity (EV-associated NLuc activity/μg of protein) of EV-associated NLuc-HSP70 was similar between the three populations (Fig. 2B). EV profiles were also similar, as judged by nanoparticle tracking analysis (Fig. 2C). On average, EVs emanating from WT, CD63KO and CD9 KO cells show a diameter of 168 + /− 71,4 nm, 156,7 + /− 73,3 nm, 170,4 nm +/− 82,1 nm, respectively.

This suggests the absence of apparent differences in EV characteristics between the three different donor cells. Note that our rough EV characterization does not rule out the possibility that the tetraspanins may be involved in the formation or composition of putative EV subtypes, but here we focus on EV-cargo delivery using a cargo-based approach. Finally, we tested the uptake and delivery capacity of EVs derived from donor WT, CD63KO and CD9KO cells and loaded on acceptor WT cells, using the aforementioned luminescence-based assay. No differences were observed, neither for the uptake, nor for the delivery (Fig. 2D, E). Altogether, these results suggest that the presence of CD63 and CD9 on the EVs is not required for internalization nor for content delivery into WT HeLa acceptor cells.

**Fig. 3:**
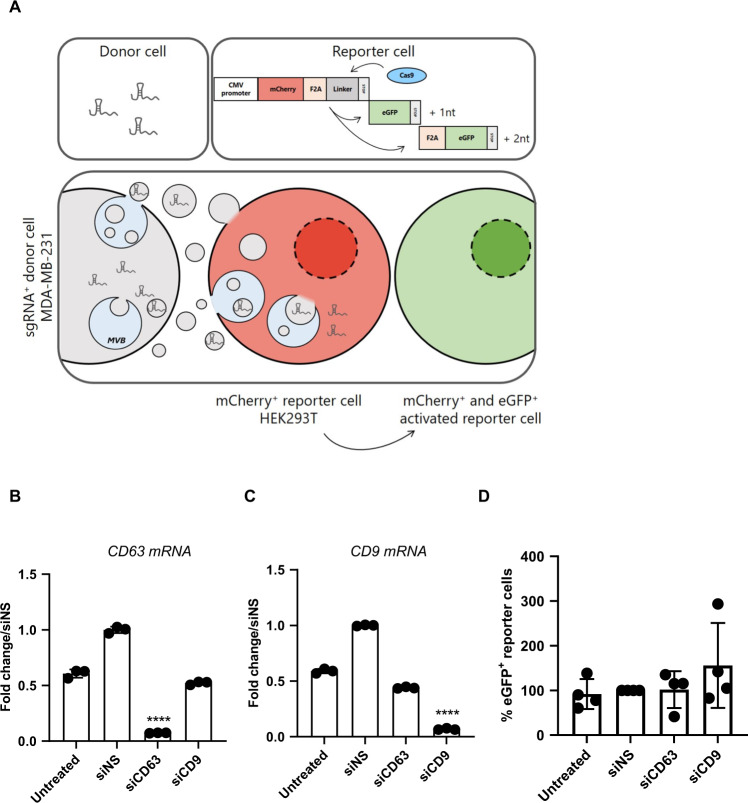
Effect of CD9/CD63 knockdown on EV acceptor cells using the CROSS-FIRE system. **A** Graphical representation of the CROSS-FIRE reporter system used to test RNA transfer efficiency. **B** CD63 and **C** CD9 gene knockdown efficiency on HEK293T acceptor cells were evaluated by RT-qPCR. Conditions were normalized against siNS and gene expression levels were normalized to GAPDH housekeeping gene levels. **D** Depletion of tetraspanins on HEK293T acceptor cells does not affect MDA-MB-231 EV-mediated RNA transfer. The number of eGFP^+^ reporter cells was determined using flow cytometry. Four independent experiments were performed, each dot represents a replicate. Error bars represent the standard deviations. **** represents *p* < 0.0001 vs siNS condition.

The same hypothesis that CD63 and CD9 are not required for EV uptake and content delivery was tested using the CROSS-FIRE system in co-culture settings (a representation of the system is depicted in Fig. 3A). For these experiments, EV-donor sgRNA^+^ MDA-MB-231 cells were used in co-culture with HEK293T Stoplight^+^ Cas9^+^ acceptor reporter cells.

First, we tested whether CD9 and CD63 on the acceptor cell’s side are required for cargo transfer. For this set of experiments reporter cells (HEK293T) were transfected with siRNAs against CD9, CD63 or a nontargeting sequence (siNS). The amount of eGFP^+^ reporter cells, and therefore the efficiency of RNA transfer, in all conditions was measured by flow cytometry after 5 days of co-culture. The efficiency of the knockdown was validated by qPCR on the reporter cells and a 93% knockdown was measured for both *CD63* and *CD9* (Fig. 3B, C). No significant change in RNA transfer efficiency was observed when either of the tetraspanins was depleted (Fig. 3D).

The presented data show that CD63 and CD9 on acceptor HEK293T cells are not required for EV (RNA) cargo transfer.

Next we set out to test whether EV uptake and cargo release are impaired in acceptor cells that receive CD9 or CD63-deficient EVs.

**Fig. 4:**
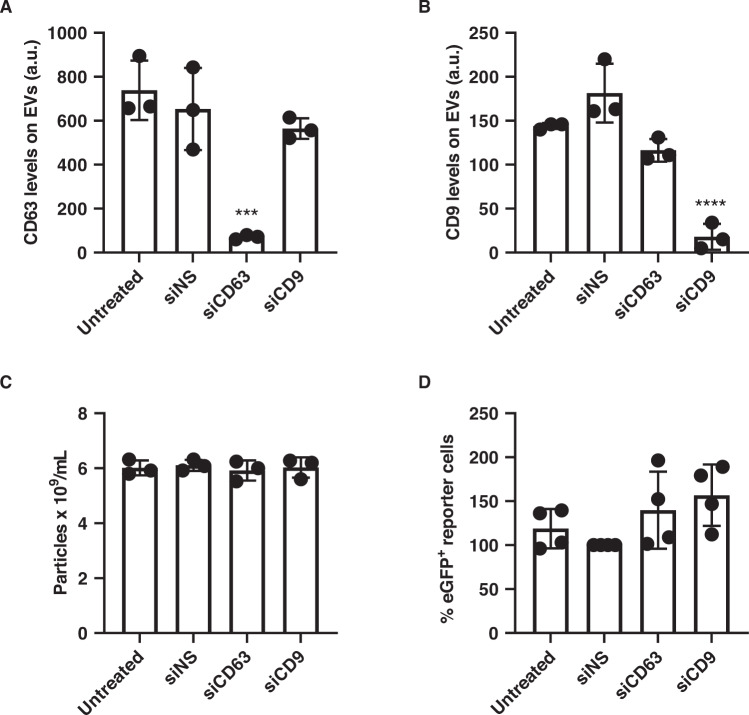
Effect of CD9/CD63 knockdown on EV release and RNA transfer using the CROSS-FIRE system. **A**, **B** Knockdown of tetraspanins in MDA-MB-231 cells results in lower levels as measured in conditioned medium. Tetraspanin levels were assessed using bead-based flow cytometry. **C** Knockdown of tetraspanins in MDA-MB-231 cells does not affect EV release. EV particle count in precleared conditioned medium was quantified using NTA. **D** Tetraspanins on MDA-MB-231 EVs do not affect EV-mediated RNA transfer to HEK293T cells. The number of eGFP^+^ reporter cells was determined using flow cytometry. Four independent experiments were performed, each dot represents a replicate. Error bars represent the standard deviations. *** represents *p* < 0.001, **** represents *p* < 0.0001 vs siNS condition.

First, we tested that siRNA-mediated knockdown of either CD63 or CD9 leads to a decrease in protein expression at the EV level. Levels of the desired EV markers were measured in precleared Opti-MEM medium collected from MDA-MB-231 donor cells previously treated with siRNAs against CD9, CD63 or a nontargeting control. Pull-down with either CD9 or CD63 magnetic beads and labelling with relevant antibodies were performed on the precleared conditioned medium to measure levels of relevant tetraspanins. Analysis of the samples by flow cytometry revealed a 89% decrease in CD63 expression (Fig. 4A) and a 90% decrease in CD9 expression (Fig. 4B). Moreover, further EV characterization by nanoparticle tracking analysis showed no significant changes in EV release upon CD9 or CD63 knockdown, as measured from the precleared conditioned medium (Fig. 4C).

We then performed a co-culture assay where donor MDA-MB-231 cells were transfected with siRNAs against CD9, CD63 or a nontargeting sequence, prior to addition of acceptor reporter HEK293T cells. The amount of eGFP^+^ reporter cells, and therefore the efficiency of RNA transfer, in all conditions was measured by flow cytometry after 5 days of co-culture. No significant change was observed when either of the tetraspanins was depleted in donor EVs (Fig. 4D).

Taken together these results confirm, with a fundamentally different assay, previous results obtained with the luminescence-based assay; that in the systems used in this study both CD63 and CD9 appear to be dispensable for EV-mediated cargo transfer.

## Discussion

Our results demonstrate that CD63 and CD9 are dispensable for EV uptake and content delivery, both in the form of protein content (as proven with the NLuc assay) and RNA content (as shown with the CROSS-FIRE assay) in the tested cell lines. We tested depletion either on donor or acceptor membranes, considering that CD63 and CD9 might have putative trans-binding partners on the opposite membranes. Our results ruled out any role of CD63/CD9 as EV-receptor localized within the acceptor membranes or receptor-ligand localized at the EV surface, or the possibility that those tetraspanins interact in-trans to promote EV-docking on the target membrane. This is consistent with the fact that the extracellular loop of the tetraspanins poorly protrudes from the membrane and they are therefore unlikely to bind ligands^18^.

When considering the effect of CD63 (and to some extent CD9) depletion on viral infection, our results might appear surprising at first sight. However, it is important to note that the mode of action of the tetraspanins on viral infection is unclear, and it remains to be clearly demonstrated if CD63 and/or CD9 are involved in virus entry/fusion or viral particle formation and release after primo-infection in macrophages and CD4^+^ lymphocytes^9^. This might be even more complex as both CD9 and CD63 have also been proposed to play an inhibitory function on virus entry^19^.

Previously, anti-CD9 antibody has been proposed to inhibit EV-uptake within acceptor cells^16,17^, suggesting a positive role of CD9 in EV uptake. This positive role was also reported in another study^20^. On the other hand, CD9 has also been proposed to be a negative regulator of EV uptake^21^. We cannot formally explain the reason of the apparent discrepancy here, but only highlight that cell models and methods were different (inhibitory antibody vs. genetic KD or KO, flow cytometry vs. luminescence or genetic modification) and only speculate that the correlation made previously between CD9 amount and EV uptake capacity might be due to an indirect signal transduction that happens within specific cells used in the other studies.

Importantly, tetraspanins make up a large protein family, and it is possible that the depletion of a single tetraspanin is compensated by redundant proteins belonging to the same family.

We do not imply here that CD63 or CD9 have no EV-related functions. It is still possible that those tetraspanins known to form tetraspanin-enriched microdomains may act as a signaling platform, or facilitate receptor/ligand interaction by bringing receptor and ligand in close spatial proximity, or participate in EV-membrane rigidity or stability, or in the formation or maintenance of the EV corona. In addition, although we did not detect changes in EVs emanating from CD63 or CD9-depleted EVs, it is still possible that tetraspanins contribute to EV architecture and composition (proteins, lipids and nucleotides). For instance, depletion of CD63 or CD9 on donor cells may alter the formation or the composition of a subpopulation of vesicles that does not contain the two cargoes that we followed in the study. Investigating the impact of CD63/CD9 depletion on a particular subtype of vesicles will require dedicated investigation. Further studies will be required to confirm our findings within more integrated and complex in vivo systems.

In summary, our results show, using two different EV uptake and cargo delivery monitoring systems, and two different approaches to gene silencing, that neither CD63 nor CD9 are required for the EV uptake and content-delivery process.

## Material and Methods

### Cell culture

HeLa cell (from ATCC, Virginia, USA) were cultured in DMEM (Gibco, Illinois, USA) complemented with 10% FBS (Biosera, France), at 37 °C 5% CO_2_. HeLa GFP-Hsp70 or NLuc-Hsp70 stable cell lines were selected with geneticin 10 μg/mL (Gibco, Illinois, USA) after lipofectamine-based transfection. CD63KO and CD9KO HeLa (gift from E Rubinstein and G Van Niel) cells were generated by CRISPR/Cas9 as described in^8^.

Except mentioned otherwise, cells were transfected using Lipofectamine 2000 (Invitrogen, Massachusetts, USA), according to manufacturer’s protocol.

sgRNA^+^ donor MDA-MB-231 and Stoplight^+^ Cas9^+^ acceptor reporter HEK293T cells were cultured in Dulbecco’s Modified Eagle Medium (DMEM) with L-Glutamine (Gibco) supplemented with 10% fetal bovine serum (FBS) (Sigma). All cell lines were cultured in the presence of 100 µg/ml streptomycin, and 100 u/ml penicillin (Gibco) at 37 °C and 5% CO2. If required, stable cell lines were cultured in medium with selection antibiotics as previously reported^4^.

### Co-culture experiments

In all co-culture experiments sgRNA^+^ MDA-MB-231 were used as donor cells and Stoplight^+^ Cas9^+^ HEK293T were used as acceptor cells. All co-culture experiments were performed in DMEM containing 10% FBS, L-Glutamine, 100 µg/ml streptomycin and 100 µ/ml penicillin. Unless stated otherwise, co-culture experiments were performed for five days at a 1:5 ratio of reporter:donor cells in a 96-well or 48-well plate setup. At the end of a co-culture experiment, cells were analyzed by flow cytometry. For flow cytometry analysis cells were trypsinized for 5 min, and transferred to a new 96-well plate using a double volume of DMEM containing 10% FBS. Cells were centrifuged for 5 min at 300 × g. Cells were then resuspended in 250 µl 1% FBS in PBS, and kept on ice until further analysis. Samples were processed on Canto or Fortessa (BD Biosciences) flow cytometers and further analyzed using FlowJo v10 software.

### siRNA knockdown

For evaluation of knockdown efficiency and co-culture experiments cells were reverse transfected and seeded in DMEM containing 10% FBS, L-Glutamine, and no antibiotics, in 48-well or 96-well plates. Cells were transfected using Lipofectamine RNAiMax (Life Technologies) according to the manufacturer’s protocol. 5 or 1.25 pmol of Dharmacon ON-TARGETplus siRNA smartpools were transfected in 48-well or 96-well plate wells, respectively. Dharmacon ON-TARGETplus siRNA smartpool products information are as follows: nontargeting control cat. no. D-001810-10; siCD9 cat. no. L-017252-00; siCD63 cat. no. L-017256-00. After 18 h, cells were washed once and subsequently cultured in DMEM containing 10% FCS, L-Glutamine, 100 µg/ml streptomycin, and 100 u/ml penicillin. For co-culture experiments, additional cells were added directly after the culture medium was changed. For qPCR analysis, cells were cultured for an additional 48 h before RNA isolation. For bead-based flow cytometry and NTA, medium was changed after 48 h and cells were cultured in Opti-MEM for further 24 h before analysis.

### Plasmids

GFP-HSP70 and NLuc-HSP70 plasmids have been described in^3^. Briefly, NLuc-Hsp70 was obtained by PCR amplifying NLuc sequence (Promega, Wisconsin, USA) prior to replacing GFP sequence from GFP-HSP70 (15215, Addgene, Massachusetts, USA) using AgeI and XhoI restriction sites.

### EV isolation

Donor HeLa cells were cultured for 24 h in serum-free DMEM. Conditioned medium was harvested and submitted to a 2000× *g* centrifugation for 20 min at 4 °C to remove cell debris, and then to a 100,000× *g* ultracentrifugation for 1 h 30 min at 4 °C (45Ti rotor, Optima^TM^ XE-90 Ultracentrifuge, Beckman Coulter, California, USA). Note that in initial experiments, intermediate step at 10,000 × *g* was performed to remove large vesicles and large protein aggregates, but this fraction was virtually depleted of proteins including EV cargo of interest (GFP-Hsp70 and NLuc-Hsp70). We subsequently removed this additional step. The obtained pellet was washed with PBS and centrifuged for 1 h at 100,000× *g* 4 °C (MLA 50 rotor with dedicated adaptors and Optima MAX-XP ultracentrifuge, Beckman Coulter, California, USA). Pellet was resuspended in 100 μL PBS and either stored at 4 °C (for up to 20 h) or immediately applied on acceptor cells. EV characterization was performed accordingly to MISEV2018 guidelines.

### Western blot

Cells were scraped on ice in DPBS and pelleted at 1000x *g* for 5 min at 4 °C. Cell pellets were resuspended in PBX lysis buffer (DPBS, Triton-X-100 1%, EDTA-free protease/phosphatase inhibitor cocktail (Roche, Switzerland)) and incubated on ice for 10 min with intermittent vortexing. Samples were then submitted to a 15,000x *g* centrifugation for 10 min at 4 °C to pellet nuclei and unbroken cells. Supernatants (cell lysates, CL) were collected. Protein concentration of cell lysate and EVs were obtained using the Micro BCA Protein Assay kit (Thermo Scientific, Illinois, USA). Samples were mixed with Laemmli buffer (Bio-Rad, France) containing 10% β-mercaptoethanol, except for CD63, and CD9 detection (no β-mercaptoethanol) and loaded on 4–15% polyacrylamide gels (Bio-Rad, France). After electrophoresis, proteins were transferred on PVDF membranes using the Trans-Blot Turbo system (Bio-Rad, France). Membranes were incubated with DPBS containing 0.05% Tween20 and 5% non-fat milk (blocking buffer), then with a 1/1000 dilution of primary antibody (α-Actin (Cat # MAB1501, Millipore, Germany), α-ALIX (Cat # 2171, Cell Signaling, Massachusetts, U.S.A.), α-Calnexin (Cat # ab133615, Abcam, U.K.), α-CD63 (Cat # 556019, BD Bioscience, New Jersey, U.S.A.), α-CD9 (Cat # cbl162, Millipore, Germany), α-Hsp70 (Cat # ADI-SPA-810-D, Enzo LifeScience, New York, U.S.A.),) in blocking buffer overnight at 4 °C. Membranes were then washed and finally incubated with a 1/5000 dilution of HRP-coupled secondary antibody (α-mouse or α-rabbit, Cat # 115-035-003, Jackson ImmunoResearch, U.K.) in DPBS containing 0.05% Tween20. The HRP signal on membranes was developed using the Clarity Western ECL substrate (Bio-Rad, France) and imaged using the ImageQuant LAS 4000 (GE Healthcare Life Sciences, France).

### Luminescence assays

For the uptake assay, HeLa acceptor cells were seeded 24 h before the uptake experiment, at 20,000 cells per well in a 96-well plate. NLuc-Hsp70 HeLa EV input was added in serum-free DMEM. Were several donor cells were tested, the amount of loaded EV was normalized according to the cargo-specific activity (luminescence activity/μg of total protein). Cells were incubated with EVs at 37 °C for 24 h, or the specifically indicated time. After incubation, cells were washed 3 times with PBS, and wells were filled with 100 mL of PBS prior adding Nano-Glo^TM^ reagent (Promega, Wisconsin, USA), following manufacturer’s instruction. Note that we used as a negative/background control empty wells, that did not contain any acceptor cells, in which we added equal amount of donor EVs for the same amount of time. After EV removal and PBS washes, luminescence was measured. This virtually-null background value was systematically subtracted from all the values of the other tested conditions.

For the Content delivery assay, acceptor cells were seeded at 200,000 cells per well in a 24-well plate and incubated with donor NLuc-H70 EVs. When required, acceptor cells were treated or not with 200 nM of Bafilomycin A1 (SML1661, Sigma-Aldrich, Missouri, USA) from 30 min before adding EV until the end of the uptake assay. Then acceptor cells were washed with PBS, detached, and collected using PBS 0.5 mM EDTA, and pelleted 10 min at 350× *g* 4 °C and resuspended in PBS 1X PPI. For detergent-free cell disruption, samples were processed as follows: 5 s vortex, 5 back-and-forth in 30 G needle and sonication (5 s, 30% amplitude) with a micro-tip sonicator (FB50, Fisher Scientist, Illinois, USA). Sample was then submitted for 10 min, 350× *g*, 4 °C, to pellet the intact cells, then the supernatant was centrifuged 1 h at 100,000× *g* 4 °C to pellet the membranes (resuspended in PBS 1X PPI) and to recover the cytosolic fraction (supernatant). NLuc activity was measured within each fraction. Luminescence activity was read using iD3 SpectraMax microplate reader (Molecular Devices, California, USA).

### Confocal Microscopy

Acceptor cells were seeded 24 h before the uptake experiment, at 200,000 cells per well in a 24-well plate on the top of coverslips. Acceptor cells were incubated with GFP-Hsp70 EVs (10 μg protein/mL) for 24 h at 37 °C). Then cells were washed with PBS, fixed 15 min RT with PBS 4% Paraformaldehyde. After washing with PBS, samples were treated with 0.2% Triton X100 and incubated with DAPI stain (Sigma Aldrich, Missouri, USA) and WGA-Alexa^633^ (Thermo Scientific, Illinois, USA) for 15 min to respectively label nucleus and plasma membrane (Supplementary Fig. 1), later delineated with cyan and magenta lines (Fig. 1D). Samples were then mounted and imaged using a SP8 confocal microscope (Leica Microsystems, Germany). WT acceptor HeLa cells not treated with fluorescent EVs, and virtually negative for GFP fluorescence, were used as negative control to set up the threshold to specifically detect GFP-foci emanating from donor EVs, when acceptor cells of interest were treated by GFP-HSP70 EVs. Fluorescent foci within cells were manually counted.

### Nanoparticle tracking analysis

EV size distribution was determined using a Nanosight S500 nanoparticle analyzer (Malvern Instruments) with a 405 nm laser. EV-containing medium (Opti-MEM) from the described conditions was precleared at 2000 x *g* for 10 min and used for measurements, with the camera setting at level 16. For post-acquisition analysis, all post-acquisition settings were set to “Auto”, with the exception of a fixed detection threshold of level 6. Using a scripted control function, five 60 s videos were recorded for each sample, and analyzed using NTA software v3.1.

### Bead-based flow cytometry on EVs

Tetraspanin expression on EVs was assessed using flow cytometry after capturing EVs from conditioned medium (Opti-MEM) precleared at 2000 x *g* for 10 min with antibody-coated magnetic beads, as described before^22^. In short, EV-containing medium was incubated overnight with either, CD9- or CD63-antibody-coated magnetic beads (ExoCap, JSR Life Sciences) and washed with 2% bovine serum albumin (BSA) in PBS. Subsequently, CD9- or CD63-Alexa647 antibody (CD9, BD Bioscience, 341648, clone M-L13; CD63, BD Biosciences, 561983, clone H5C6) in PBS was added and incubated for 2 h at RT while shaking. After washing with 2% BSA in PBS, samples were resuspended in 0.25% BSA in PBS for analysis. The mean fluorescence intensity (MFI) of bead-captured EVs was measured using flow cytometry (BD FACSCanto II).

### Cell RNA isolation and qPCR analysis

Total RNA was isolated from cells using TRIzol Reagent (Thermo Fisher Scientific), according to the manufacturer’s protocol. Isolated RNA was measured using a DS-11 Spectrophotometer (DeNovix). 300 ng RNA per sample were used for cDNA synthesis using the iScript cDNA Synthesis Kit (Bio-Rad). qPCR was performed using iQ SYBR Green Supermix (Bio-Rad) in a CFX96 Real‐Time PCR Detection System (Bio‐Rad). Primer sequences were synthesized by Integrated DNA Technologies. Cycle threshold (Ct) values were normalized per experiment and per gene. ΔΔCt was calculated using the housekeeping gene GAPDH.

Primer sequences: CD9 Fw TTCCTCTTGGTGATATTCGCCA, Rev AGTTCAACGCATAGTGGATGG; CD63 Fw CAGTGGTCATCATCGCAGTG, Rev ATCGAAGCAGTGTGGTTGTTT; GAPDH Fw ACAGTCAGCCGCATCTTC, Rev GCCCAATACGACCAAATCC.

### Statistics and reproducibility

Experiments were performed in 2–4 independent replicates. The data are presented as mean ± SD. Statistical analysis with more than two groups was performed with one-way analysis of variance (ANOVA) followed by Dunnett’s multiple comparisons test. All statistical analyses were performed with Prism software (Graphpad prism for windows, version 9.0). Differences were considered significant at *P  <  0.05, **P  <  0.01, ***P  <  0.001, **** *p* < 0.0001.

### Reporting summary

Further information on research design is available in the Nature Portfolio Reporting Summary linked to this article.

## Supplementary information

Supplementary Information

Description of Additional Supplementary Files

Supplementary Data

Reporting Summary

## Data Availability

The data generated during this study are provided in the main paper (Figs. 1–4), and Supplementary Figs. 1–2. Single data-points used to generate graphs are available in the Supplementary Data file. Uncropped blots have been included in Supplementary Fig. 2. Any additional information required is available from the corresponding authors.
