# Supplementary material for: Lack of involvement of CD63 and CD9 tetraspanins in the extracellular vesicle content delivery process
Source: Commun Biol. 2023 May 17;6:532. doi: 10.1038/s42003-023-04911-1 (PMC10192366; doi:10.1038/s42003-023-04911-1)
Supplement: Supplementary file 1 — Supplementary Information [file 42003_2023_4911_MOESM1_ESM.pdf]

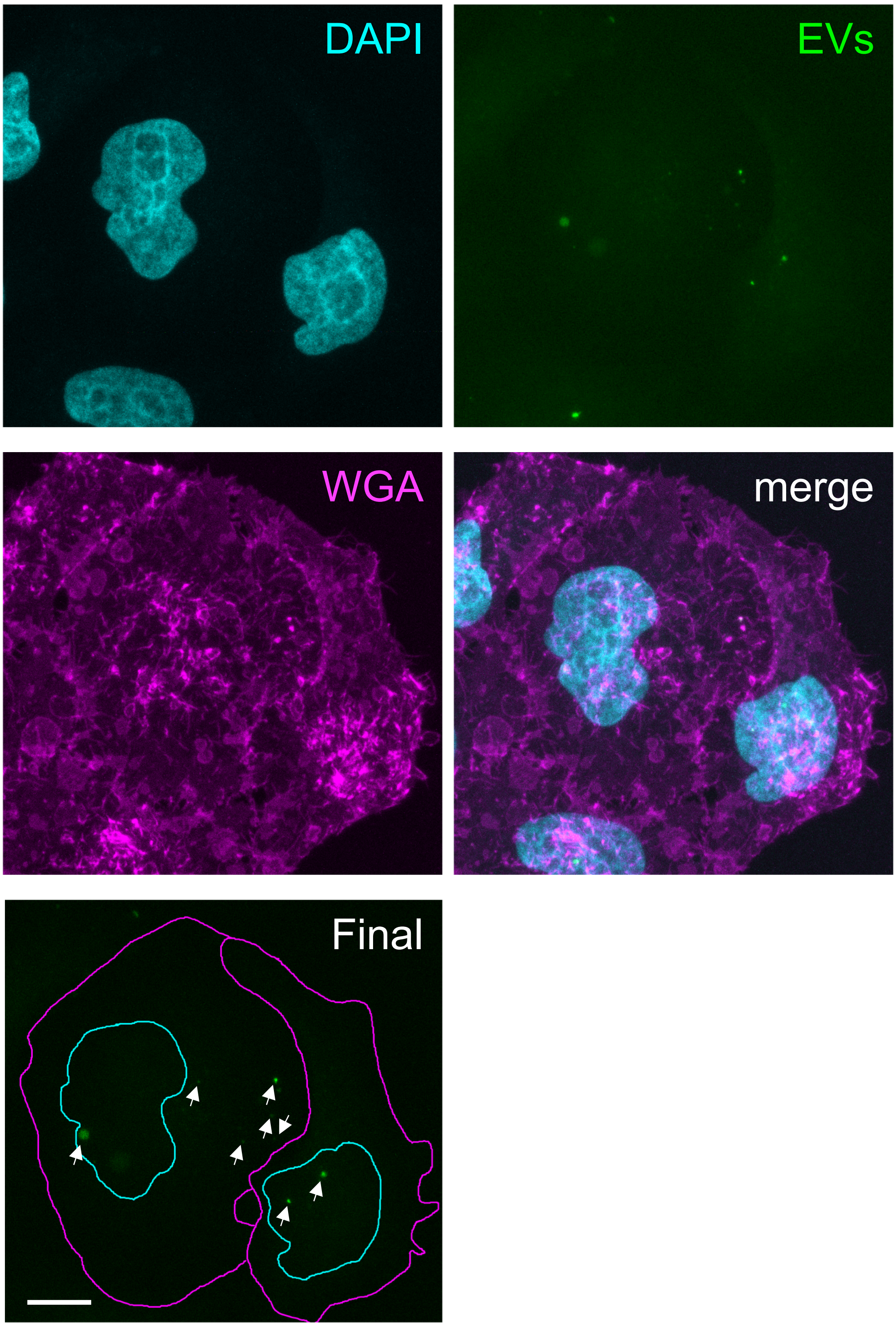

Supplementary Figure 1. To visualize Plasma and Nuclear Membranes, cells (treated with GFP-HSP70 positive EVs) were labeled with DAPI and WGA^{Alexa633}. For illustration purposes, nuclei and PM were then outlined with cyan and purple lines, as shown in Figure 1D. Sale bar, 10 μm.

Figure 1A.

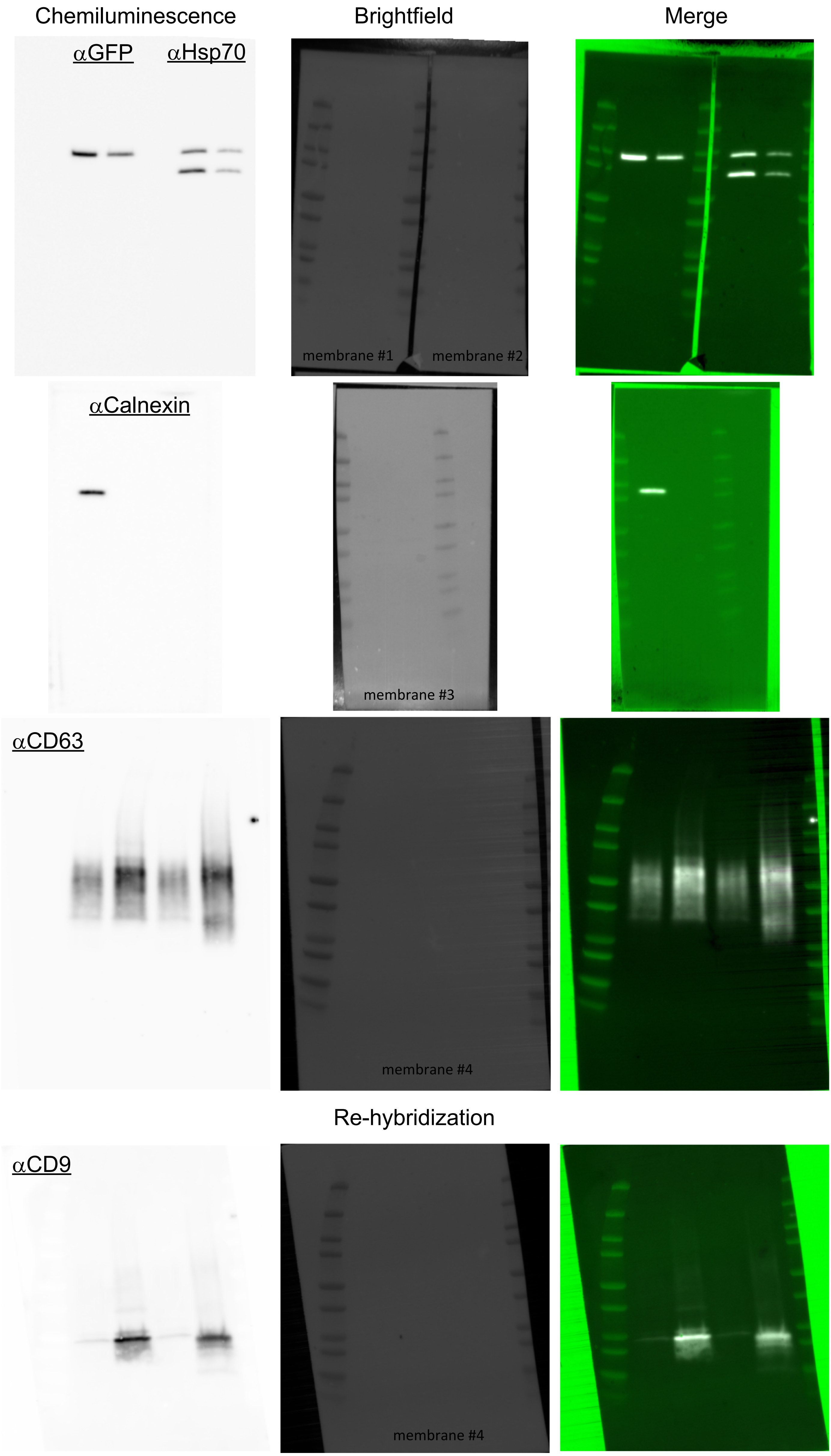
Chemiluminescence
Brightfield
Merge
αGFP
αHsp70
membrane #1
membrane #2
αCalnexin
membrane #3
αCD63
membrane #4
Re-hybridization
αCD9
membrane #4

Figure 1B.

Chemiluminescence

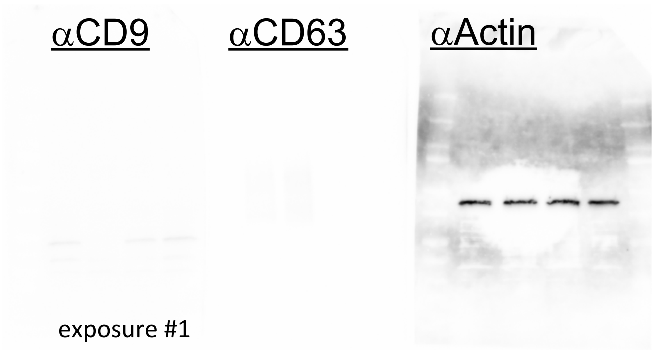

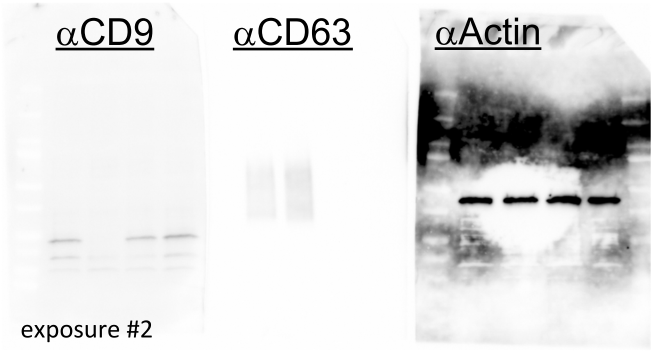

Brightfield

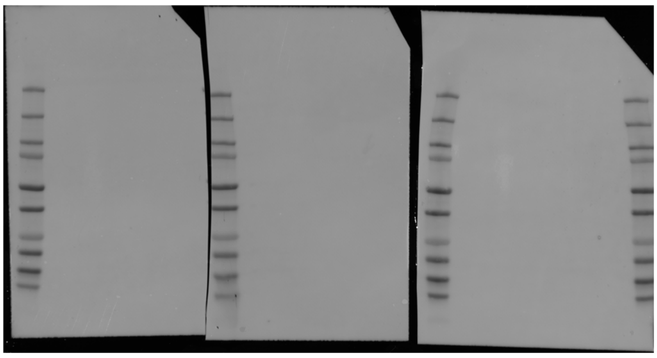

Merge

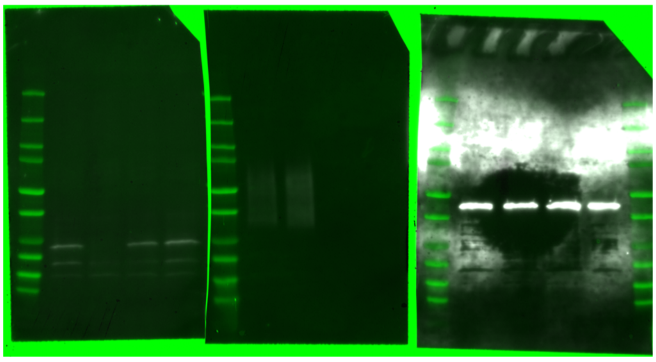

Figure 2A.

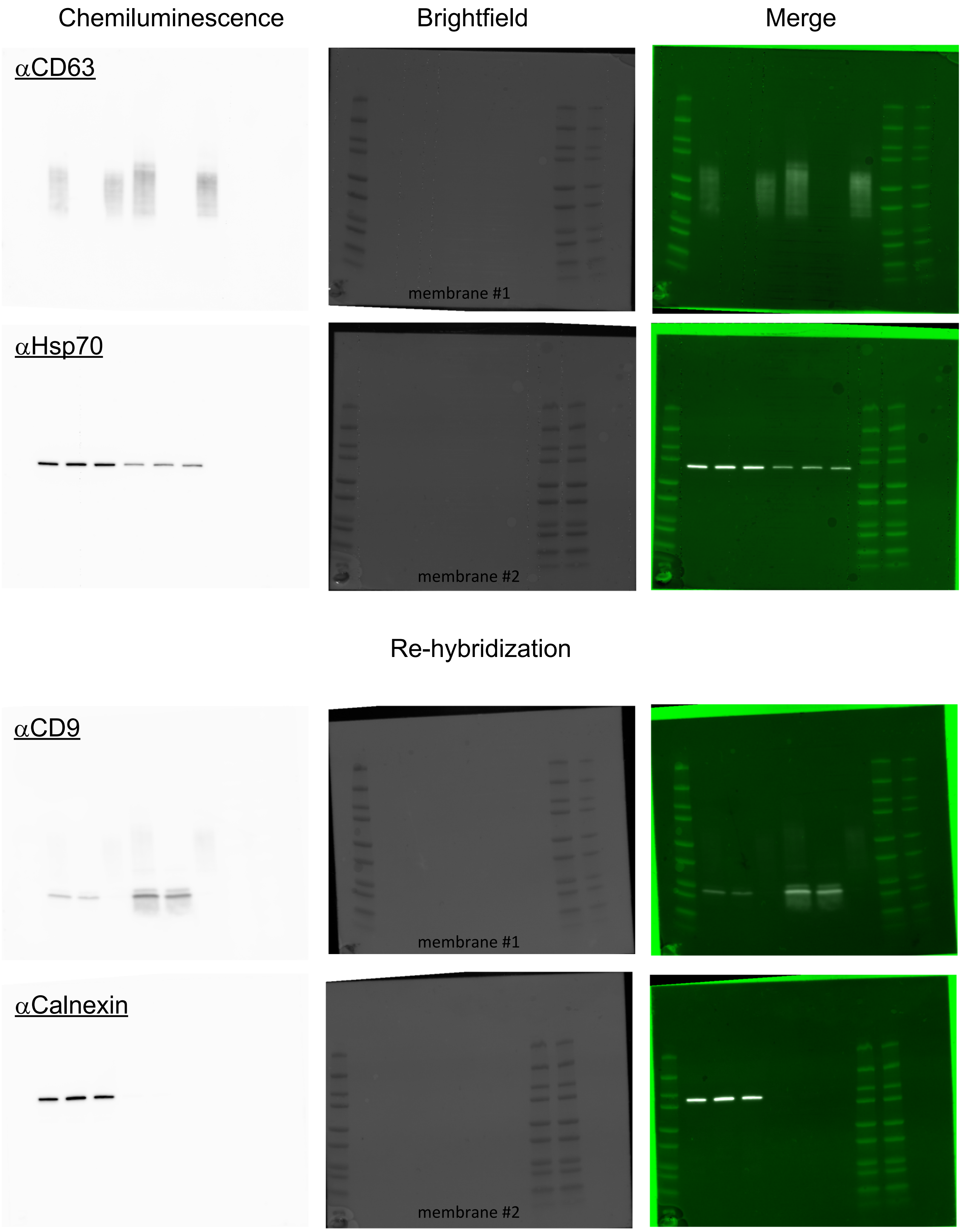

Supplementary Figure 2: Original and Uncropped pictures (HRP signal, brightfield and overlay) of all western blots shown in the study. Related figures areindicated above each panels.
