# Supplementary material for: Lack of involvement of CD63 and CD9 tetraspanins in the extracellular vesicle content delivery process
Source: Commun Biol. 2023 May 17;6:532. doi: 10.1038/s42003-023-04911-1 (PMC10192366; doi:10.1038/s42003-023-04911-1)
Supplement: Supplementary file 2 — Description of Additional Supplementary Files [file 42003_2023_4911_MOESM2_ESM.pdf]

Description of Additional Supplementary Files

File name: Supplementary Data
Description: Single data-points used to generate graphs.
