# Supplementary material for: Lack of involvement of CD63 and CD9 tetraspanins in the extracellular vesicle content delivery process
Source: Commun Biol. 2023 May 17;6:532. doi: 10.1038/s42003-023-04911-1 (PMC10192366; doi:10.1038/s42003-023-04911-1)
Supplement: Supplementary file 4 — Reporting Summary [file 42003_2023_4911_MOESM4_ESM.pdf]

nature portfolio

Corresponding author(s): Gregory Lavieu, Pieter Vader

Last updated by author(s): Apr 18, 2023

Reporting Summary

Nature Portfolio wishes to improve the reproducibility of the work that we publish. This form provides structure for consistency and transparency in reporting. For further information on Nature Portfolio policies, see our Editorial Policies and the Editorial Policy Checklist.

Statistics

For all statistical analyses, confirm that the following items are present in the figure legend, table legend, main text, or Methods section.

n/a	Confirmed	
☐	☒	The exact sample size (*n*) for each experimental group/condition, given as a discrete number and unit of measurement
☐	☒	A statement on whether measurements were taken from distinct samples or whether the same sample was measured repeatedly
☐	☒	The statistical test(s) used AND whether they are one- or two-sided<br>*Only common tests should be described solely by name; describe more complex techniques in the Methods section.*
☒	☐	A description of all covariates tested
☐	☒	A description of any assumptions or corrections, such as tests of normality and adjustment for multiple comparisons
☐	☒	A full description of the statistical parameters including central tendency (e.g. means) or other basic estimates (e.g. regression coefficient) AND variation (e.g. standard deviation) or associated estimates of uncertainty (e.g. confidence intervals)
☐	☒	For null hypothesis testing, the test statistic (e.g. *F*, *t*, *r*) with confidence intervals, effect sizes, degrees of freedom and *P* value noted<br>*Give P values as exact values whenever suitable.*
☒	☐	For Bayesian analysis, information on the choice of priors and Markov chain Monte Carlo settings
☒	☐	For hierarchical and complex designs, identification of the appropriate level for tests and full reporting of outcomes
☒	☐	Estimates of effect sizes (e.g. Cohen's *d*, Pearson's *r*), indicating how they were calculated

Our web collection on statistics for biologists contains articles on many of the points above.

Software and code

Policy information about availability of computer code

Data collection	Data was collected using up-to-date software provided by the manufacturer for all the equipment listed in the Methods section.
Data analysis	Flow cytometry data was analyzed using FlowJo v10, statistical analyses were performed using Graphpad Prism 9. Nanoparticle tracking analysis measurements were analyzed using NTA software v3.1.

For manuscripts utilizing custom algorithms or software that are central to the research but not yet described in published literature, software must be made available to editors and reviewers. We strongly encourage code deposition in a community repository (e.g. GitHub). See the Nature Portfolio guidelines for submitting code & software for further information.

Data

Policy information about availability of data

All manuscripts must include a data availability statement. This statement should provide the following information, where applicable:

- Accession codes, unique identifiers, or web links for publicly available datasets
- A description of any restrictions on data availability
- For clinical datasets or third party data, please ensure that the statement adheres to our policy

The datasets generated during and/or analysed during the current study are available from the corresponding author on reasonable request.
the authors declare that the data supporting the findings of this study are available within the supplementary information files. Further requests should be addressed to the corresponding authors. Source data are provide with this paper

Human research participants

Policy information about studies involving human research participants and Sex and Gender in Research.

Reporting on sex and gender	N/A
Population characteristics	N/A
Recruitment	N/A
Ethics oversight	N/A

Note that full information on the approval of the study protocol must also be provided in the manuscript.

Field-specific reporting

Please select the one below that is the best fit for your research. If you are not sure, read the appropriate sections before making your selection.

☒ Life sciences ☐ Behavioural & social sciences ☐ Ecological, evolutionary & environmental sciences

For a reference copy of the document with all sections, see nature.com/documents/nr-reporting-summary-flat.pdf

Life sciences study design

All studies must disclose on these points even when the disclosure is negative.

Sample size	No sample size-calculation was performed prior to experiments, as no preliminary data was available on effect size and variation. Experiments were performed 2 - 4 times, and analyzed using the statistical analyses listed throughout the manuscript. In no case were 1-sided statistical tests performed. For flow cytometry, an average of 30000 events/sample were analyzed.
Data exclusions	One out of four technical replicates in one biological replicate of co-culture experiments was excluded due to outstandingly high reporter cell percentage, compared to the other 3 technical replicates. no other data were excluded.
Replication	Experiments were performed 2-4 times, number of replicate is formally indicated in the figure legends.
Randomization	Samples were not randomized during this study. It is unlikely that this has affected results, as all samples were measured at the same time using the same machine settings (eg. laser voltage, camera settings, etc...). Moreover, all samples were analyzed using the exact same gates, analytical formulas and settings.
Blinding	Samples were not blinded as all samples were measured at the same time using the same machine settings (eg. laser voltage, camera settings, etc...). Moreover, all samples were analyzed using the exact same gates, analytical formulas and settings

Reporting for specific materials, systems and methods

We require information from authors about some types of materials, experimental systems and methods used in many studies. Here, indicate whether each material, system or method listed is relevant to your study. If you are not sure if a list item applies to your research, read the appropriate section before selecting a response.

Materials & experimental systems

n/a	Involved in the study	
☐	☒	Antibodies
☐	☒	Eukaryotic cell lines
☒	☐	Palaeontology and archaeology
☒	☐	Animals and other organisms
☒	☐	Clinical data
☒	☐	Dual use research of concern

Methods

n/a	Involved in the study	
☒	☐	ChIP-seq
☐	☒	Flow cytometry
☒	☐	MRI-based neuroimaging

Antibodies

Antibodies used	As described in the Methods section: 1/1000 dilution of primary antibody (α-Actin (Cat # MAB1501, Millipore, Germany), α-ALIX (Cat

Antibodies used	# 2171, Cell Signaling, Massachusetts, U.S.A.), α-Calnexin (Cat # ab133615, Abcam, U.K.), α-CD63 (Cat # 556019, BD Bioscience, New Jersey, U.S.A.), α-CD9 (Cat # cbl162, Millipore, Germany), α-Hsp70 (Cat # ADI-SPA-810-D, Enzo LifeScience, New York, U.S.A.)
Validation	all antibodies were validated prior this study and/or already published (bonsergent et al, Nat Comm 2021, Bonsergent et al, FEBS letter , 2019)

Eukaryotic cell lines

Policy information about cell lines and Sex and Gender in Research

Cell line source(s)	HEK293T and HeLa cells were ordered from the ATCC.<br>MDA-MB-231 cells were kindly provided by dr. SJ Vervoort and Prof. dr. P. Coffer from the UMC Utrecht (as used in: eLife, 2018, Global transcriptional analysis identifies a novel role for SOX4 in tumor-induced angiogenesis.). Original source: ATCC.
Authentication	Cell lines were authenticated by morphological assessment through light microscopy.
Mycoplasma contamination	All cell lines were tested negative for mycoplasma.
Commonly misidentified lines<br>(See ICLAC register)	No commonly misidentified lines were used in this study.

Flow Cytometry

Plots

Confirm that:

☒ The axis labels state the marker and fluorochrome used (e.g. CD4-FITC).

☒ The axis scales are clearly visible. Include numbers along axes only for bottom left plot of group (a 'group' is an analysis of identical markers).

☒ All plots are contour plots with outliers or pseudocolor plots.

☒ A numerical value for number of cells or percentage (with statistics) is provided.

Methodology

Sample preparation	As described in the Methods section for co-culture experiments: "For flow cytometry analysis cells were trypsinized for 5 min, and transferred to a new 96-well plate using a double volume of DMEM containing 10% FBS. Cells were centrifuged for 5 min at 300×g. Cells were then resuspended in 250µl 1% FBS in PBS, and kept on ice until further analysis. Samples were processed on Canto or Fortessa (BD Biosciences) flow cytometers and further analyzed using FlowJo v10 software."<br><br>As described in the Methods section for bead-based FC: "In short, EV-containing medium was incubated overnight with either, CD9- or CD63-antibody-coated magnetic beads (ExoCap, JSR Life Sciences) and washed with 2% bovine serum albumin (BSA) in PBS. Subsequently, CD9- or CD63-Alexa647 antibody (CD9, BD Bioscience, 341648, clone M-L13; CD63, BD Biosciences, 561983, clone H5C6) in PBS was added and incubated for 2 h at RT while shaking. After washing with 2% BSA in PBS, samples were resuspended in 0.25% BSA in PBS for analysis. Mean fluorescence intensity (MFI) of bead-captured EVs was measured using flow cytometry (BD FACSCanto II)."
Instrument	Canto or Fortessa (BD Biosciences) flow cytometers
Software	BD Diva 8 software and FlowJo v10 software
Cell population abundance	For flow cytometry, an average of 30000 events/sample were analyzed.
Gating strategy	For analyses performed with Fortessa flow cytometers (co-culture):<br>Firstly, cell debris was gated out and cells were selected using FSC and SSC.<br>Then, single cells were selected by plotting SSC-A vs SSC-H.<br>Then, donor and reporter cell populations (Stoplight- vs Stoplight+) were selected by mCherry signal using the Yellow-Green laser. For the reporter cells (mCherry+ gated), reporter activation was assessed by measuring eGFP expression using a blue (488 nm) laser. eGFP+ cells were counted as activated reporter cells.<br>For all analyses: gating settings were set based on signals in all measured channels using donor cells (mCherry-eGFP-), untreated reporter cells (mCherry+eGFP-) and targeting sgRNA transfected reporter cells (mCherry+eGFP+). The same gates were used for all samples and conditions within the same experiment.

☐ Tick this box to confirm that a figure exemplifying the gating strategy is provided in the Supplementary Information.
